# Controlled nano-agglomerates as stabile SERS reporters for unequivocal labelling

**DOI:** 10.1038/s41598-022-12989-6

**Published:** 2022-05-28

**Authors:** Can Xiao, Bernat Mir-Simón, Pilar Rivera-Gil

**Affiliations:** Integrative Biomedical Materials and Nanomedicine Lab, Department of Medicine and Life Sciences, University Pompeu Fabra, Doctor Aiguader 88, 08003 Barcelona, Spain

**Keywords:** Nanoscale materials, Cell biology

## Abstract

Biosensors, especially those with a SERS readout, are required for an early and precise healthcare diagnosis. Unreproducible SERS platforms hamper clinical SERS. Here we report a synthetic procedure to obtain stabile, reproducible and robust highly-SERS performing nanocomposites for labelling. We controlled the NPs agglomeration and codification which resulted in an increased number of hot spots, thus exhibiting reproducible and superior Raman enhancement. We studied fundamental aspects affecting the plasmonic thiol bond resulting in pH exhibiting a determining role. We validated their biosensing performance by designing a SERS-based detection assay model for SARS-CoV-2. The limit of detection of our assay detecting the spike RBD was below 10 ng/mL.

## Introduction

The design for precise diagnosis is critical to human health as for preventing pandemics or other biothreads. Nanosystems has been widely developed in sensor devices for diagnostics, in vitro and in vivo diagnosis^1^. This diagnosis field takes benefits from the design and synthesis of nanomaterials^2–4^, especially noble metal nanomaterials showing localized surface plasmon resonance (LSPR) properties. This LSPR phenomenon limits nanomaterials absorbing specific region of light, and makes the nanomaterials sensitive to the modifications of physical properties of nanomaterials and their environments showing plasmonic absorption shifts^5–7^. Based on their optical properties, flexible functionalized nanomaterials have already been applied in sensing varieties of biomolecules, including biomarkers for cancer^8^, enzyme^9^, DNA^10^, and other biological species^11^.

Surface enhanced Raman spectroscopy (SERS) also depends on plasmonic platforms^12^. SERS signals can be collected from the molecules which are in close proximity of nanometallic surfaces with confined LSPR^13^. In general, average enhancement factors for typical SERS substrates are amplified by 10^6^ to 10^8^ orders of magnitude comparing with their Raman signature^14^. Moreover, this enhancement can be further increased up to 10^15^ orders of magnitude to SERS signal by hot spots^15^, which are caused by the plasmonic coupling of the particles when they are very close to each other^14^. Thus, hot spots have critical importance when designing a SERS nanostructure. Gold and silver are the most commonly applied materials for SERS substrates, as they offer high field enhancement in the visible to near infrared range due to their high density of electrons^16^. SERS as a non-destructive technology provides chemical information in aqueous environments. Thus SERS has been an effective tool to realize qualitative and quantitative detection of biological species^12,17–19^, including micro RNA analysis^20^, enzyme^21^, hydrogen peroxide^22^, staphylococcal enterotoxin B^23^ and other diseases biomarkers^24,25^ with gold nanowire, gold-silver alloy NPs and gold-MnO_2_ core–shell, hollow gold nanospheres and gold nanostar and nanosphere separately. However, producing homogenous, sensitive and reproducible SERS platforms is the main difficulty which hampers SERS bioapplications^26^, with many efforts made in the design and synthesis of uniform nanomaterials^27–29^. The design and controllable synthesis of nanostructures are critical steps towards implementing SERS in medicine^12^.

Here we present a controllable design and synthesis of a nanostructure confining encoded silver nano-agglomerates inside silica coating (AgNPs@MBA@SiO_2_). We systematically studied the fundamental aspects and optimized the thiol silver bonding for encoding silver nanoparticles. With the help of controlled agglomeration, we improved the percentage of hot spots which guaranteed the extremely high Raman enhancement. These encoded agglomerates were further encapsulated with a silica shell to protect them from oxidation, contaminations and increase the stability for a long period of time thanks to the unique properties of the SiO_2_ layer (*e.g.,* surface chemistry, biocompatibility, optical transparency, and colloidal stability). With further biofunctionalization with antibody, we also demonstrated the performance of our devices for SARS-CoV-2 detection. The reference diagnosis for SARS-CoV-2 is based on reverse transcription polymerase chain reaction (RT-PCR)^30^. Yet there are false positive or negative reports, especially for the early stages. Biosensors as alternative or supplementary solutions are being developed based on plasmonic nanomaterials. Gold nanoparticles have been developed for colorimetric detection of SARS-CoV-2 with isolated RNA samples^31^ and for IgM Antibodies against the SARS-CoV‑2 virus detection based on a lateral flow device^32^. A more sophisticated devises based on gold nanoislands was reported for SARS-CoV-2 detection targeting selected sequences^33^. Compared with the biosensors mentioned, we selected the detection of SARS-CoV-2 spike RBD protein, which is the major immunodominant protein^34^. One of the promising advantages of our biosensor is that it can potentially screen SARS-CoV-2 without prior sample treatment. This whole designed procedure provides our nanostructure high Raman enhancement and robust intensity for sensitive sensing, and uniform synthesis for stability and repeatability. We offered an optimized, stabile nanostructure which can be reliably applied in biosensing.

## Methodology

A detailed description of the methodology and additional results are presented in the supporting information file.

### AgNPs@MBA@SiO_2_ synthesis

In briefly, we performed a bottom up synthesis of AgNPs relying on the chemical reduction of metal salts and controlling the shape and size by citrate reduction of AgNO_3_^35^. Then, we co-adsorbed and covalently bound onto the metallic AgNPs surfaces a SERS probe 4-mercaptobenzoic acid (MBA) and a polymeric stabilizer CTPEG12. Finally, we controlled their agglomeration and encapsulation within a homogenous layer of silica following a modification of the Stöber method^36^.

### SERS based biosensing

AgNPs@MBA@SiO_2_ were biofunctionalized with SARS-CoV/SARS-CoV-2 Spike antibody (AgNPs@MBA@SiO_2_@Ab) via GPTMS. Then SERS-based biosensing followed a sandwich-based diagnostic assays as described in detail in supporting information. The limit of detection (LOD) was calculated by measuring the intensity at 1075 cm^−1^ with the presence and the absence of antigen, with a 3:1 ratio threshold (Signal/Noise = 3).

## Results and discussion

### AgNPs@MBA@SiO_2_ synthesis and characterization

**Figure 1:**
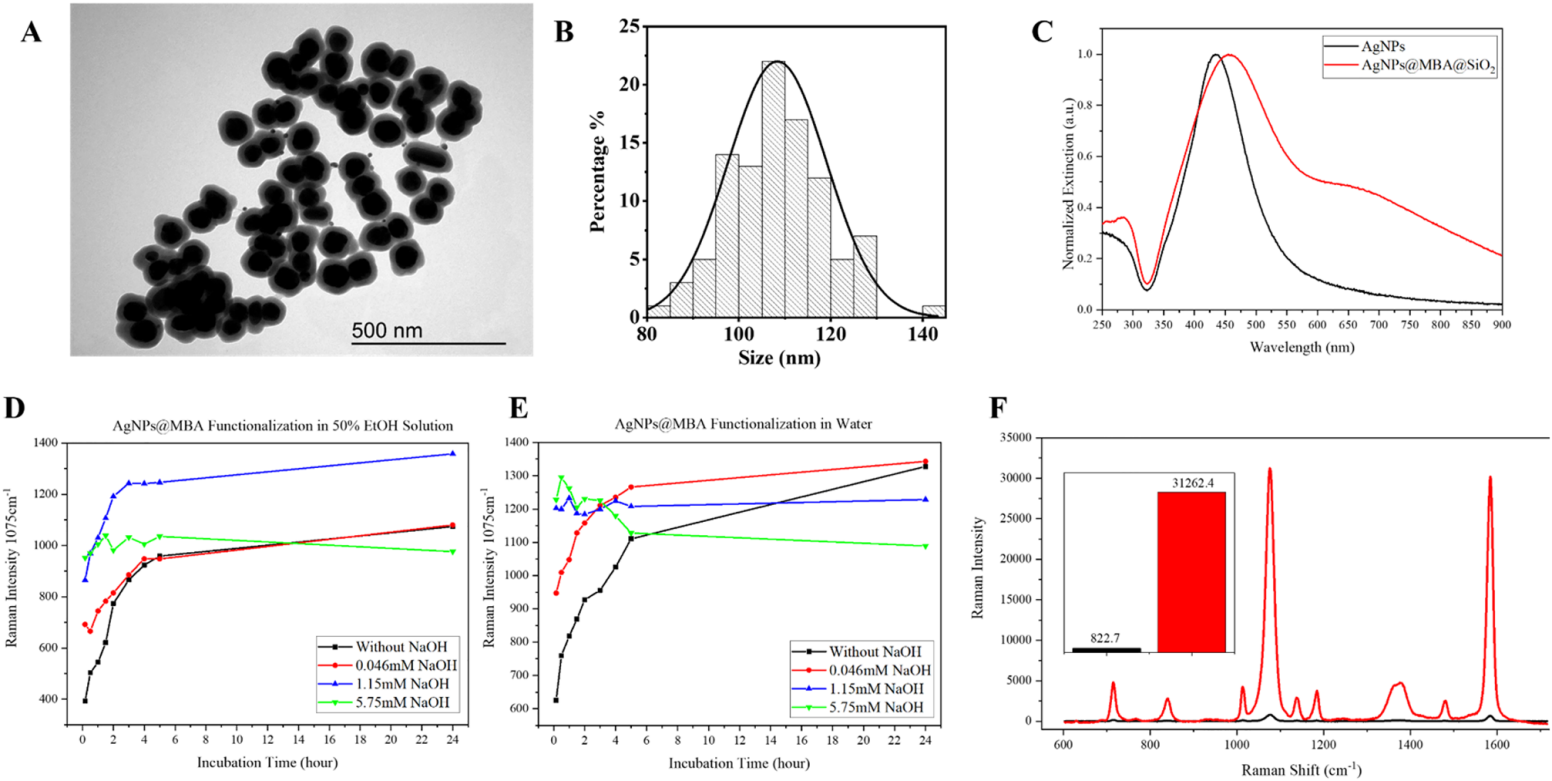
Synthesis and characterization of the SERS-responsive nanoagglomerates. (**A**) TEM image of AgNPs@MBA@SiO_2_. (**B**) Size distribution of AgNPs@MBA@SiO_2_ based on the quantification of 100 NPs from TEM images. (**C**) UV–Visible extinction spectra of AgNPs (black) and AgNPs@MBA@SiO_2_ (red). (**D**,**E**) MBA adsorption kinetic under different conditions (with or without NaOH) measured by SERS intensity at 1075 cm^−1^ (**D**) in 50% EtOH/water solution and (**E**) in aqueous solution. Black line: without NaOH; red line: with 0.046 mM NaOH; blue line: with 1.15 mM NaOH; green line: with 5.75 mM NaOH. (**F**) SERS spectra and SERS intensity at 1075 cm^−1^ (inset image) of non-agglomerated AgNPs@MBA@SiO_2_ (in black), and nanoagglomerates, AgNPs@MBA@SiO_2_ (in red).

We have synthesized a SERS encoded core–shell nanostructure comprising silver NPs agglomerates. Figure 1A shows the geometry of AgNPs@MBA@SiO_2_. The plasmonic encoded nanoagglomerates are within a silica layer. This layer offers unique properties (*e.g.,* surface chemistry, biocompatibility, optical transparency, and colloidal stability). As a result, it protects the nanoagglomerates from oxidation and signal-contamination, thus ensuring a long-term SERS signal stability. The AgNPs were spherical with an anisotropy aspect ratio close to 1. We analysed more than 100 AgNPs@MBA@SiO_2_ from the TEM images. Around 1% of the particles were non-spherical, *i.e.,* rod-shaped particles or quasi-flat triangles. More than 60% of the agglomerates were isolated dimers, trimers, tetramers, pentamers, hexamers, or mixtures thereof. Each NP have four different angles for size measurement. The AgNPs core was approx. 60–70 nm and the SiO_2_ layer was around 20 nm. The complete size of AgNPs@MBA@SiO_2_ was around 110 nm (Fig. 1B) and their hydrodynamic diameter 133.8 nm (PDI 0.130) with a zeta potential of -24.7 mV (cf., SI, Fig. S1). Further characterization of the NPs was performed with the UV–Vis spectrum^37^. Figure 1C shows the normalized extinction spectra of the individual, citrate capped AgNPs and the nanoagglomerates, AgNPs@MBA@SiO_2_. The characteristic spherical AgNPs’ LSPRs is centred around 430 nm. As for AgNPs@MBA@SiO_2_, it appears a new prominent shoulder absorption at around 700 nm. This red-shift and broadening of the LSPR to higher wavelengths confirmed the formation of nanoagglomerates.

Aromatic compounds are commonly used as SERS or Raman probes as they have high Raman cross section^12,17^. Previous reported articles used ethanol as solvent during Raman probe modification based on the consideration of low solubility of hydrophobic Raman probes^38^. However, to the best of our knowledge, there is still lack of detailed information on the fundamental factors affecting Raman probes NPs’ modification. Therefore, we studied the formation of thiol-silver bond as a function of pH and solvent within this work. We used CTPEG12 to stabilize the system during the modification under different pHs. The SERS probe used here is an aromatic molecule MBA which has high Raman cross section^18,39^ and the polymeric stabilizer CTPEG12 is an aliphatic chain polymer with 12 carbon atoms and a carboxylic group at the end of the chain. Both MBA and CTPEG12 were bonded to AgNPs through their thiol group by forming covalent bonds.

To study the pH and solvent effect on MBA adsorption, we monitored the evolution of the characteristic ring breathing band of adsorbed MBA at 1075 cm^−1^ over the time^40^ and under different conditions (Fig. 1D,E). AgNPs@MBA were dispersed in 50% EtOH/water solution (Fig. 1D and cf., SI, Fig. S2) or in aqueous solution (Fig. 1E and cf., SI, Fig. S3) containing no or different amount of NaOH from 10 min to 24 h. Water and ethanol have different physical property (*e.g.,* pKa and dielectric constant), therefore, it was not surprising to measure different pH values for each of the solvents even with the same amount of NaOH^41^ (cf., SI, Table S1).

Figure 1D,E shows the adsorption and bonding kinetic results of MBA onto the AgNPs under the different conditions (pH and solvent) and over time. When comparing the first 10 min after the addition of Raman code in both solvents, the intensity at 1075 cm^−1^ increased along with the system pH and amount of added NaOH. The codification reached a plateau intensity in a pH dependent manner being shorter under increased alkaline conditions. The adsorption of thiol onto silver mental surface follows physisorption to chemisorption where the S–H bond breaks and forms silver thiol covalent bond^42^. Environmental pH will affect the deprotonation of thiol group, thus will affect the formation speed of silver thiol bond. As we observed in our system, an alkaline environment facilitates the codification of MBA onto silver metallic surfaces. When comparing the plateau intensity after 24 h, the number of adsorbed MBA were similar when the codification was performed in 50% ethanol/water solution with 1.15 mM NaOH (pH 8) and in aqueous solution with 0.046 mM NaOH (pH 8.5). These conditions exhibited the maximum SERS intensity among all. Subsequent additions of NaOH and pH increase resulted in a MBA codification decrease in both solvents. Considering that the pKa of MBA and CTPEG12 is around 4–5^43^, the deprotonated MBA and CTPEG12 are both negatively charged. This electrostatic repulsion on the surface and the competition between MAB and CTPEG12 will hamper MBA bonding from the bulk solution, since in general, compared with aromatic thiols, aliphatic thiols have better electrochemical and thermodynamic stability^44^. Dissociation was also favored at higher pH^45^, which could also be related to this dynamic equilibrium. Ethanol has little effect on the modification and stability of metal-thiol bond. Although it was demonstrated that because of the reduction effect of ethanol on gold, the strength of metal-thiol contacts can be weakened^46^, ethanol is still recommended for hydrophobic Raman probes.

The number of MBA molecules adsorbed on the metallic surface and the packing of the monolayer is discussed within this work. We show that they depend on factors like the metal-thiol bonding formation speed, which is affected by pH, the electrostatic repulsion on the metallic surface, and the competition between Raman probe and stabilizer. Controlling the pH in reaction is especially critical to achieve optimized codification of metallic surfaces with thiolated aromatic compounds.

By controlling the agglomeration of AgNPs@MBA and the MBA adsorption, we managed to increase the SERS efficiency up to nearly 40 times when comparing the SERS spectra (characteristic peak intensity at 1075 cm^−1^) of AgNPs@MBA@SiO_2_ and non-agglomerated AgNPs@MBA@SiO_2_ (Fig. 1F). This high SERS response was attributed to active SERS structures called “hot spots”^14^. In comparison to aggregation or uncontrolled agglomeration (cf., Fig. S5), where the NPs merged without hot spots^38^, controlled agglomeration offers a useful tool to rationally design nanomaterials for SERS labelling.

Herewith, we report a universal protocol for synthesizing stabile SERS encoded core–shell nanostructure. We used the same protocol for other plasmonic nanoestructures like gold (cf., SI, Fig. S6) and for other Raman probes^47^.

### Stability of the AgNPs@MBA@SiO_2_ SERS signal after labelling different types of substrates

**Figure 2:**
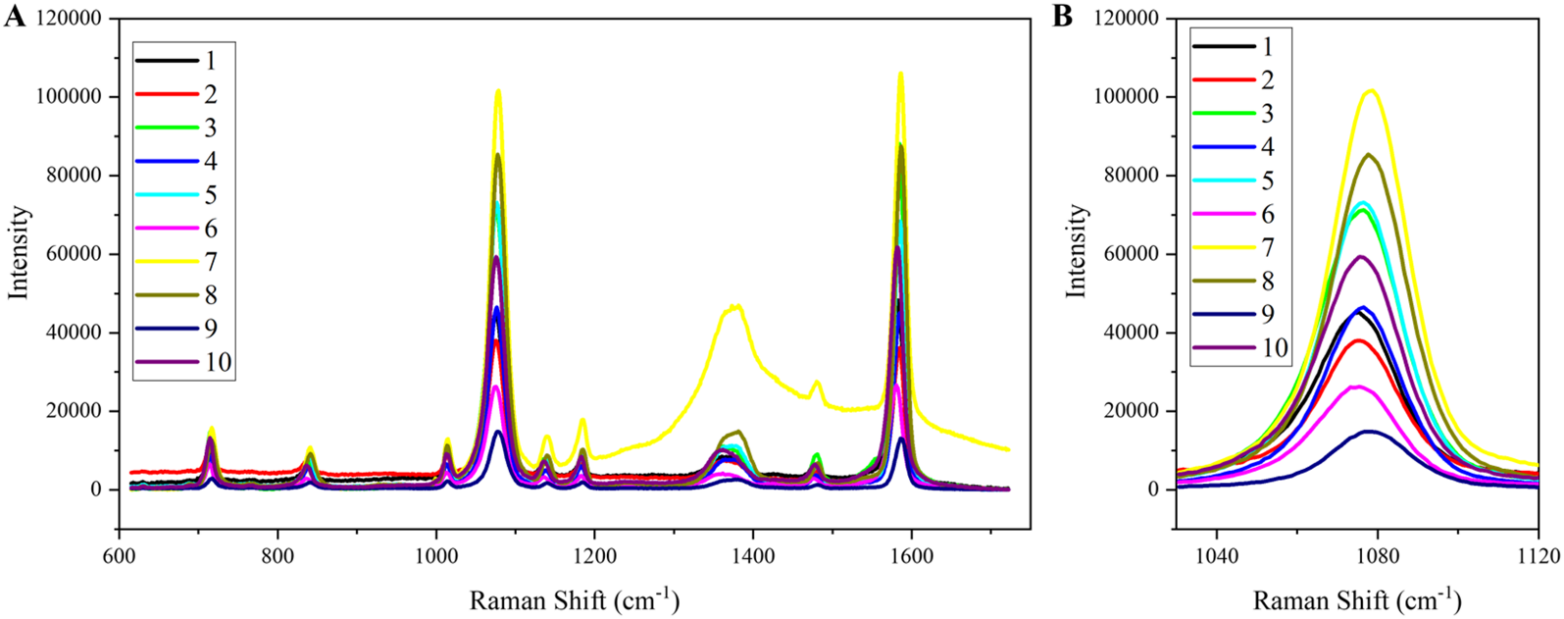
SERS characterization of different substrates wherein the AgNPs@MBA@SiO_2_ has been placed. (**A**) SERS spectra and (**B**) zoomed spectra showing characteristic peak at 1075 cm^−1^ of AgNPs@MBA@SiO_2_ on different materials: 1, semi-aniline leather; 2, aniline leather; 3, pigmented leather; 4, polyester; 5, silk; 6, plastic (PVC); 7, glass; 8, brass; 9, cotton; and 10, dyed pigmented leather.

We analysed the SERS signal robustness and stability of our AgNPs@MBA@SiO_2_ by depositing them onto 10 different substrates made of different materials (cf., SI, Fig. S7). Figure 2 shows corresponding SERS spectra and zoomed characteristic MBA peak at 1075 cm^−1^ of AgNPs@MBA@SiO_2_ demonstrating efficient labelling of all substrates. Except for glass which shows a broad band between 1000 and 1800 cm^−1^, the background signal originated from all substrates was neglectable. Regardless, both effects did not affect the characteristic bands of MBA at 1075 cm^−1^ which is used for tracing and labelling. We did observe an uneven SERS intensity between all substrates that can be explained by differences in the distribution of AgNPs@MBA@SiO_2_ within the different substrates.

**Figure 3:**
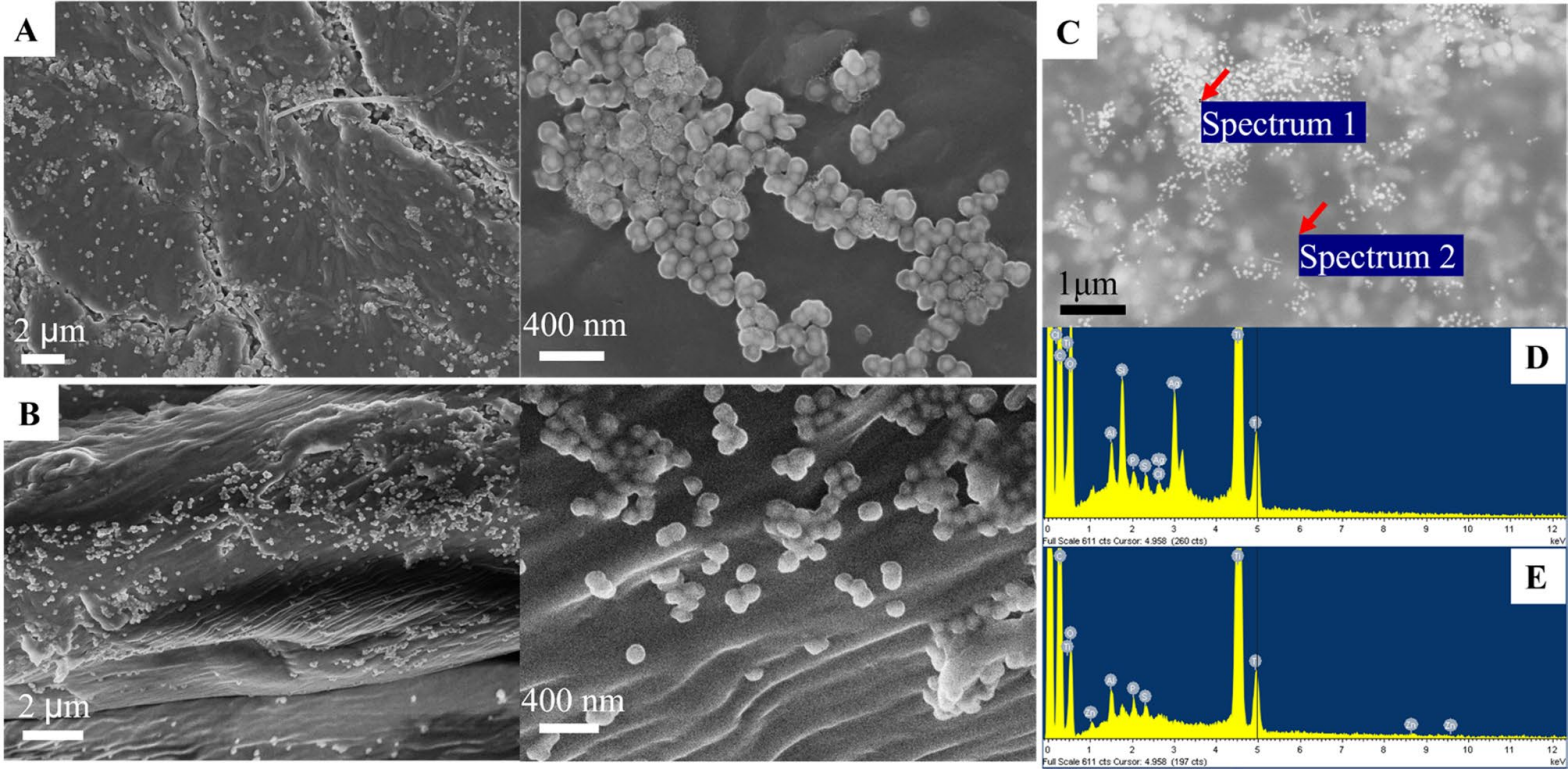
Scanning Electron Microscopy (SEM) and elemental analysis (EDX) of the materials labelled with AgNPs@MBA@SiO_2_. SEM images of leather (**A**) or cotton (**B**) labelled with AgNPs@MBA@SiO_2_ showing their distribution within the substrate depending on their characteristics (**C**) SEM image of (**A**) showing areas with (spectrum 1) and without (spectrum 2) visible NPs that were selected for elemental analysis. (**D**,**E**) Their corresponding spectra. The peaks from left to right in (**D, spectrum 1**) correspond to: Cl, C, Ti, O, Al, Si, P, S, Ag/Cl, Ag, Ti, Ti, and in (**E, spectrum 2**) to: C, Ti, O, Zn, Al, P, S, Ti, Ti.

We decided to examine the AgNPs@MBA@SiO_2_ distribution within the different substrates and its impact on the NPs’ geometry (Fig. 3). For example, in the case of cotton (Fig. 3B), the NPs are deposited onto the cotton fibres and hidden within the fibres, whereas in the leather (Fig. 3A), the NPs were more localized in the labelling spot (zoomed SEM areas of Fig. 3A,B). SEM images shows that the distribution can vary with surface properties of the substrates like roughness and porosity. Distribution of the NPs within the substrate can influence the SERS signal. Indeed, we observed that the SERS signal for the dye pigmented leather was higher than for cotton (Fig. 2). The leather is less rough and porous than cotton, thus hindering the diffusion of the AgNPs@MBA@SiO_2_ through the substrate and enhancing the SERS signal. At any rate, both substrates demonstrated preservation of the NPs’ geometry, thus SERS signal despite the different distribution. For the elemental analysis (Fig. 3D,E), we took two areas of the labeled spot on the leather (Fig. 3C), one with visible AgNPs@MBA@SiO_2_ (spectrum 1) and another with no visible NPs (spectrum 2). Figure 3D confirms the presence of trace elements from the AgNPs@MBA@SiO_2_.

AgNPs@MBA@SiO_2_ exhibit robust SERS signals; obtained in 0.1 s and with insignificant interfere by other components of the substrate. This provides our SERS-responsive NPs with numerous potential applications where high optical signal is needed. The application field ranges from industrial applications (labelling and tracing of goods) where a robust, labelling signal is required or tracing to clinical biosensing and environmental sciences.

Within this work, we demonstrate the capabilities of our AgNPs@MBA@SiO_2_ to enhance the sensitivity of immunoassays for example, to detect immunogenic SARS-CoV-2 spike RBD protein, responsible for COVID-19 pandemics.

### SERS-based SARS-CoV-2 spike RBD diagnosis

**Figure 4:**
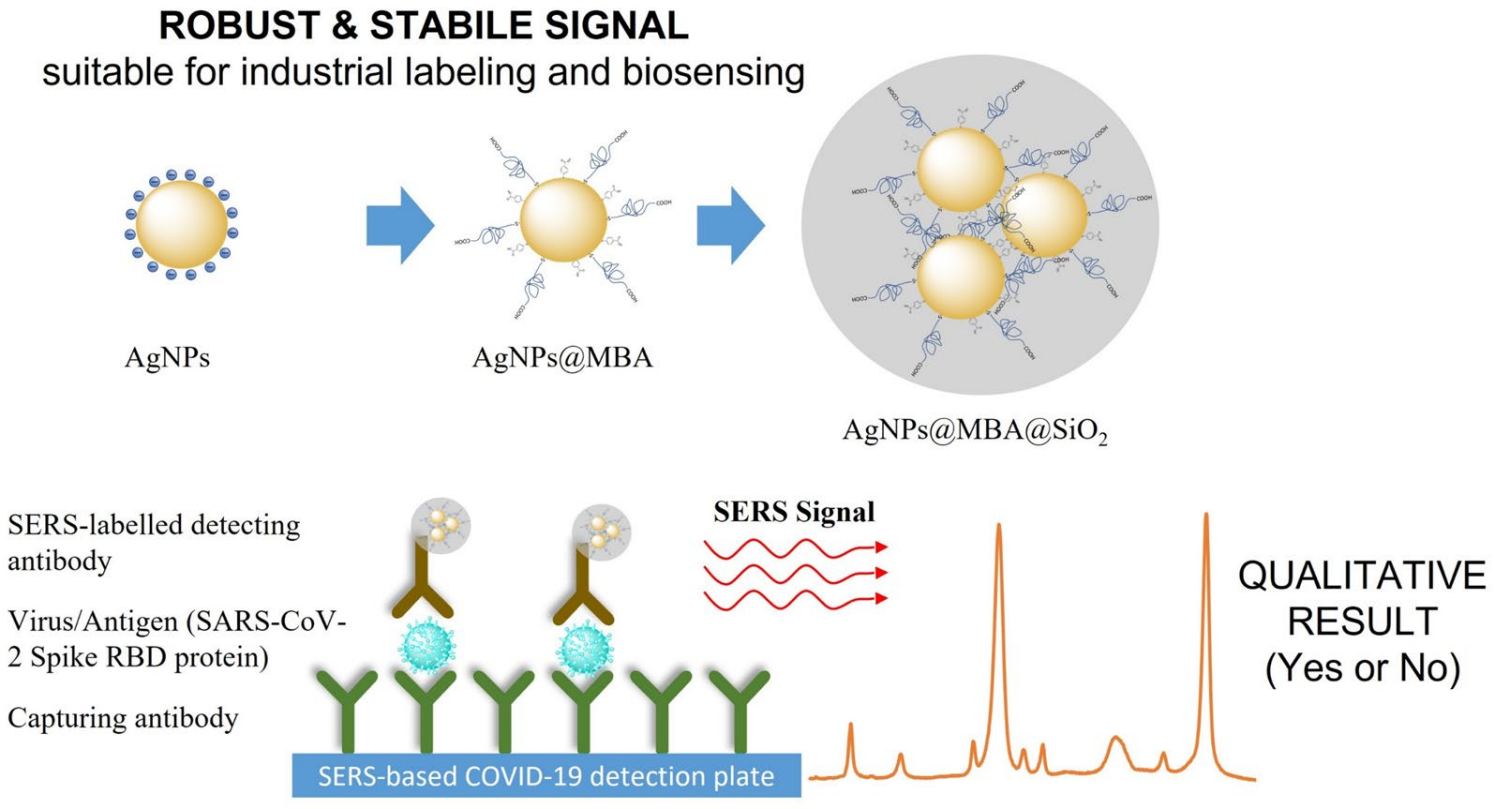
Scheme of the rational and controlled synthesis of robust and stabile SERS encoded plasmonic-silica nanocomposites for enhanced, fast, sensitive, and selective labeling and biosensing.

Herewith, we prepared our SERS-based diagnostic kit followed a sandwich-based assay, consisting of a substrate functionalized with capture antibody, antigen for diagnosis and signal amplification system. In our case, the readout is the SERS signal provided by the AgNPs@MBA@SiO_2_ which recognizes the antigen upon their surface functionalization with the antibody. Figure 4 shows a scheme of the technology.

For the surface functionalization of the AgNPs@MBA@SiO_2_ with the antibody, we first attached a linker (GPTMS) through a reaction called epoxy-silanization^48^. It enriches the NPs’ surface with epoxy groups which are highly active towards amino groups. Thus, forming covalent bonds between antibodies and GPTMS-NPs. The obtained AgNPs@MBA@SiO_2_@Ab recognize the immunogenic spike RBD protein (cf., SI, Fig. S9).

**Figure 5:**
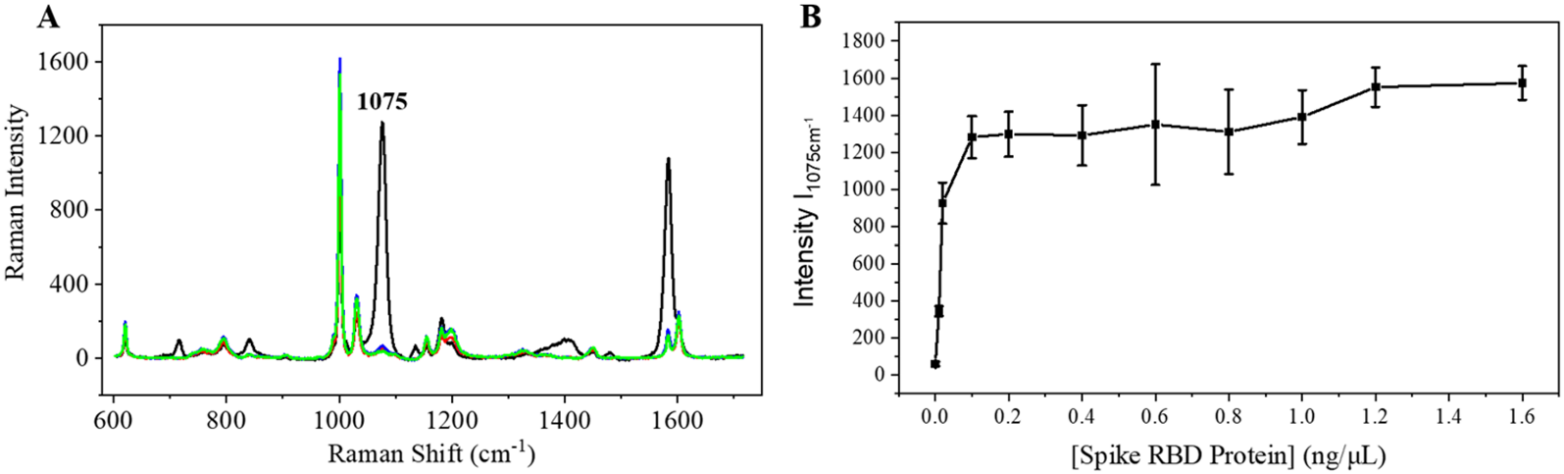
Functional validation of our SERS-based diagnostic test. (**A**) Averaged SERS spectra collected from AgNPs@MBA@SiO_2_@Ab in the presence of 100 ng/mL antigenic spike RBD protein (black) and in the absence of antigen (red), capturing antibody (blue) or both (green). In the absence of capturing antibody, the concentration of antigen was increased to 1 μg/mL. (**B**) SERS intensity at 1075 cm^−1^ provided by the AgNPs@MBA@SiO_2_@Ab incubated with different amount of antigenic spike RBD protein (from 0 to1.6 μg/mL). The results correspond to an average of 8 spectra recorded from 8 random places in the plate.

Once we engineered the detecting antibody with a SERS readout, we prepared the plate by coating the substrate with a SARS-CoV-2 spike antibody. Upon addition of the spike RBD protein and rinsing the unbounded molecules, we added our AgNPs@MBA@SiO_2_@Ab. The NPs recognize the antigen protein via a second recognition site and are immobilized onto the plate substrate. This recognition provides a positive SERS signal (enhancement of the 1075 cm^−1^ band) thus confirming virus detection (Fig. 5). The signal was fast (1 s) and specific for the viral protein. Neither the plate (cf., SI, Fig. S9) nor the absence of antigenic spike RBD protein (Fig. 5A, red) or/and the absence of capturing antibody (Fig. 5A, blue, green respectively) provided a SERS positive signal. In the absence of capturing antibody, we increased the concentration of antigen one order of magnitude to detect possible unspecific interactions of the antigen with the plate that could be responsible for false positive results, but we did not detect signal. Only when the viral antigen was immobilized, the AgNPs@MBA@SiO_2_@Ab provided a strong SERS enhancement of the specific band at 1075 cm^−1^. The enhancement was 20 times higher than in the control samples.

We correlated the concentration of AgNPs@MBA@SiO_2_@Ab with the amount of SARS-CoV spike RBD protein (Fig. S10). We selected a concentration of 9 × 10^9^ NPs/mL for all experiments because the signal was highest while maintaining the sensitivity. We further conducted the tests with a concentration series of the antigen ranging from 0 to 1.6 μg/mL (Fig. 5B). The intensity ratio between 10 and 0 ng/mL at 1075 cm^−1^ was close to 6, revealing that the LOD of AgNPs@MBA@SiO_2_@Ab for antigenic spike RBD protein detection should be lower than  10 ng/mL.

We want to mention that when the antigen concentration is higher than 100 ng/mL, the response is less sensitive to small changes in analyst’s concentration (Fig. 5B). However, the specificity is maintained since we obtained positive signals all over the antigen concentration range. A reduction in the sensitivity, can be neglected in the cases where a qualitative response is required, like for example for the fast diagnosis of viral infections.

Other SARS-CoV-2 assays with different readouts than SERS have demonstrated to be extremely specific and sensitive. For example, S. Kim et al., reported electrical transduction of the spike protein onto a bilayer quasi-freestanding epitaxial graphene with immunodetection concentrations as low as 1 ag/ml^49^. S. Mavrikou et al*.*, presented a cell-based diagnostic kit where the readout is the change in the cellular bioelectric properties measured upon membrane binding of the spike protein by bioelectric recognition assay (LOD 1 fg/mL)^50^. L. Jong-Hwan et al*.*, reported a lateral flow immunoassay detecting 10 ng/mL (1 ng/reaction) SARS-CoV-2 RBD (predicted MW 26.54 kDa). The novelty of this assay is to report for the first time, matched antibody pairs for SERS-immunoassay of SARS-CoV-2 antigens^51^. Table S2 shows a comparison between different SARS-CoV-2 spike protein detection assays recently reported. The table shows the biomarker, the readout, the material composition, LODs and the references. 3 out of 7 exhibited higher sensitivity with complicated geometries and/or not-intuitive signal readouts. In this regard, we would like to remark, the easy handling of our system in terms of sample preparation and signal interpretation for non-experts. Our SERS encoded nanocomposites, AgNPs@MBA@SiO_2_@Ab, offer a robust and stabile labelling platform that can be applied for example in sandwich-based diagnostic assays. Our immunoassay qualitatively detects SARS-CoV Spike/RBD protein (predicted MW 26.54 kDa) with high sensitivity (10 ng/mL (0.5 ng/reaction)) and specificity due to the SERS readout. Besides, other industrial (non-biomedical) applications like for example counterfeiting or product traceability are foreseen.

## Conclusion

We have rationally designed a universal^47^ and stabile synthetic procedure SERS encoded plasmonic-silica nanocomposites for a robust and enhanced signal. We have studied fundamental factors affecting the codification of plasmonic NPs with SERS probe for an enhanced labelling efficiency. In general, weak basic environment facilitates the codification of Raman probe onto metallic surface. Continuous increase of the surrounding pH will favour the dissociation of the metallic-thiol covalent bond. Thus, a precise control over the pH during the codification is critical for an optimized modification of thiolated compound onto metallic surfaces. We also found that ethanol has little effect on the formation of silver thiol bond. The final packing quality of the Raman probe monolayer is affected by the metal-thiol bonding formation speed, the electrostatic repulsion on the metallic surface, and the competition between Raman probe and stabilizer. A precise control over the NPs agglomeration, increases the percentage of hot spots which results in a SERS signal increase of nearly 40 times. This encoded nanoagglomerates were encapsulated and protected by a silica layer which offers the possibility to be multifunctionalized. The nanoaggomerates provide a robust and stable SERS signal with no interference regardless the substrate used. The range of applicability of these nanoagglomerates goes from industrial labelling to environmental science and clinical biosensing, where a fast, in situ, sensitive and specific readout is required. We validated the performance of our nanoagglomerates for the qualitative diagnosis of COVID-19 using the spike RBD. We built up a SERS-based diagnostic model kit for the fast, sensitive, and specific detection of SARS-CoV-2 antigens.

## Supplementary Information

Supplementary Information.
