# Supplementary material for: Controlled nano-agglomerates as stabile SERS reporters for unequivocal labelling
Source: Sci Rep. 2022 May 28;12:8977. doi: 10.1038/s41598-022-12989-6 (PMC9142785; doi:10.1038/s41598-022-12989-6)
Supplement: Supplementary file 1 — Supplementary Information. [file 41598_2022_12989_MOESM1_ESM.pdf]

Controlled nano-agglomerates as stabile SERS reporters for unequivocal labelling

Can Xiao[a], Bernat Mir de Simón[a,*], Pilar Rivera-Gil[a,*]

[a] Integrative Biomedical Materials and Nanomedicine Lab, Department of Experimental and Health Sciences, University Pompeu Fabra, Doctor Aiguader 88, 08003 Barcelona, Spain.

* corresponding authors: bernat.mir@upf.edu ; pilar.rivera@upf.edu

SUPPORTING INFORMATION

EXPERIMENTAL SECTION

Materials and Reagents

Ammonia solution, Tetraethyl orthosilicate, Silver nitrate ($AgNO_3$), Gold(III) chloride hydrate, Magnesium sulfate ($MgSO_4$), Ascorbic acid, 4-Mercaptobenzoic acid, Sodium hydroxide, carboxy-PEG12-thiol (CTPEG12), Ethanol, (3-Glycidyloxypropyl) trimethoxysilane (GPTMS), Ammonium Sulfate, Bovine Serum Albumin, HBSS, PBS, EIA/RIA 96 well plate, hydrogen peroxide (H_2O_2, 50wt.% in water), hydrochloric acid (HCl), nitric acid (HNO_3), were purchased from Merck. tri-Sodium Citrate 2-Hydrate (Na_3Cit) was purchased from PanReac Applichem. SARS-CoV/SARS-CoV-2 Spike antibody, Chimeric Mab (Cat: 40150-D001 and Cat: 40150-D003) and SARS-CoV Spike/RBD Protein (Cat: 40150-V08B2) were purchased from Sino Biological. Alexa Fluor 488 AffiniPure Goat Anti-Mouse IgG (H+L) was purchased from JacksonImmuno. All the chemicals were used without further purification.

Silver and gold nanoparticles synthesis

$AgNO_3$ 0.1 M, $MgSO_4$ 0.1 M, Na_3Cit 0.1 M and Ascorbic acid 0.1 M were prepared in aqueous solution. Solutions were prepared and used freshly. To avoid contamination, the glassware and magnets used were cleaned with Aqua Regia, basic piranha (RCA) before synthetizing silver nanoparticles (AgNPs) and gold nanoparticles (AuNPs).

For synthesizing AgNPs, two solutions were prepared separately. Solution 1 is prepared by mixing 5.115 mL 0.1 M Na_3Cit solution with 375 µL 0.1 M Ascorbic acid solution and solution 2 is prepared by mixing 839 µL 0.1 M $MgSO_4$ solution and 1.116 µL 0.1 M $AgNO_3$ solution. Two solutions were prepared at the same time when 250 mL Milli Q water was already boiled in an Erlenmeyer flask under homogeneous and strong stirring. Solution 1 was first added in one shot into this boiling aqueous solution after prepared for 4 min. Then solution 2 was added in one shot after one more minute. The mixture was kept stirring at 300 ºC for 30 minutes. Then the solution was cooled down at room temperature without stirring. The nanoparticles (NPs) were protected from the light and stored at 4 ºC.

For synthesizing AuNPs, 250 mL Milli-Q water was heated in an Erlenmeyer flask at 300 ºC under homogeneous and strong stirring. Once water started boiling, 678 µL 0.1 M Na_3Cit added after water boiled. 2 min later, 623.7 µL 0.1 M $HAuCl_4$ added in one shot. Reaction was kept at 300 ºC for 30 min under stirring. Then the NPs was kept undisturbed and cooled down to room temperature, and further stored at 4 ºC and protected from the light.

Modification and controllable agglomeration of AgNPs and AuNPs

The modification and controllable agglomeration procedure applied for both AgNPs and AuNPs.

AgNPs or AuNPs synthesized were firstly cleaned by centrifugation 5400 rpm (2500 g) 20 min and adjusted to approx. 2.9×10^{10} NPs/mL with Milli-Q water calculated by UV-vis extinction. Raman probe MBA and stabilizer CTPEG12 were prepared in ethanolic solution with 10^{-3} M concentration and stored in 4 ºC. CTPEG12 was responsible for the NPs' stabilization independent from the environmental pH. CTPEG12 and MBA amounts

were calculated based on the metallic surface of the NPs which would be used for modification. 1 molecule/nm^2 of the CTPEG12 and 3 molecules/nm^2 of MBA added to certain volume of ethanol under vigorous stirring. Equal volume of cleaned NPs (2.9×10^{10} NPs/mL) aqueous solution were added to this alcoholic solution under strong stirring after 5 min of the MBA and CTPEG12 addition. Then fresh prepared NaOH solution with final concentration 1.15 mM was added to NPs mixture. Reaction was kept under stirring for 24 h to finish modification.

MBA modified AgNPs and AuNPs were agglomerated in a controlled manner by two centrifugation steps. First centrifugation at 4800 rpm (2000 g) 20 min and a second centrifugation at 2000 rpm (350 g) 15 min. NPs were redispersed with Milli-Q water.

The SERS spectra of MBA modified AgNPs agglomeration (AgNPs@MBA) were collected with a Renishaw's inVia Qontor Raman system equipped with a Leica confocal microscope. The spectrograph used a high-resolution grating (1200 l cm^{-1}), band-pass filter optics, a NIR laser (785 nm) and a Peltier cooled CCD array detector, equipped with Windows-based Raman Environment (Wire™) software. 4.55×10^{10} NPs/mL (calculated by UV-vis extinction) 200 µl of AgNPs@MBA solution was added into 96 well plate for SERS spectra acquisition. The laser was focused into the samples with an 5X objective (NA 0.12), providing a laser spot diameter of approximate 8 µm. The spectra were collected with 1 s exposure time and 100 mW laser power at the samples.

SiO_2 encapsulation

Silica encapsulation was conducted in the same way for agglomerated and non-agglomerated MBA modified AgNPs and agglomerated and non-agglomerated MBA modified AuNPs.

304.7 µl of NH_4OH (35%) were added into 15 mL of EtOH and mixed properly, followed by adding 2.3 mL (approx. 7×10^{10} NPs/mL) of modified NPs in aqueous solution, and mixing the whole system properly. 12.4 µl of TEOS 10% v/v diluted by ethanol were added consecutively into this mixture, and mixing the solution by stirring for 30 seconds. Then leave the reaction system undisturbed for approx. 12 h. Silica coated modified NPs were cleaned by centrifugation thrice (6000 rpm, 20 min). Samples were stored at 4 ºC and protected from light.

The morphology of silica encapsulated MBA modified AgNPs and AuNPs agglomerations (AgNPs@MBA@SiO_2 and AuNPs@MBA@SiO_2) were checked with transmission electron microscopy (TEM), using a JEOL JEM 1010 TEM operating at an acceleration voltage of 80 kV with a tungsten filament. For the preparation of TEM samples, 10 µL of AgNPs@MBA@SiO_2 or AuNPs@MBA@SiO_2 ethanolic solution were dropped on a TEM grid. TEM samples were completely dry at room temperature before we started TEM analysis. The morphology was checked and the average size and polydispersity was calculated with at least 100 particles by using image process software "Image J".

Dynamic light scattering (DLS) and zeta potential measurements were performed with Malvern Zetasizer Nano ZS. Aqueous samples were transferred into disposable polystyrene cuvette for size measurements and disposable folded capillary cells for zeta potential measurements. Each sample was measured 3 repeats.

The extinction spectrum of each synthetic intermediate was recorded with Ultrospec™ 2100 pro UV-Visible spectrophotometer. 600 μL diluted aqueous samples were added into a Quartz cuvette (104-002-10-40, Hellma), and the extinction spectra between 250 and 900 nm wavelength were collected with Milli-Q water as a reference.

Evaluating the labelling robustness of AgNPs@MBA@SiO_2 on different substrates

20 μl 7×10^{10} NPs/mL of AgNPs@MBA@SiO_2 were dropped on different materials and the composition was left to dry at room temperature. The materials used here are listed as following: semi-aniline leather, aniline leather, pigmented leather, polyester, silk, plastic (PVC), glass, brass, cotton, pigmented leather.

SERS characterization of all samples prepared here were conducted using Renishaw inVia Qontor Raman. 20X Leica objective was used with integration time 0.1 s and a power at the sample of 3 mW.

The surface of AgNPs@MBA@SiO_2 deposited materials were analyzed with a scanning electron microscope (SEM) from Phenom XL Desktop SEM with a Backscattered electron detector. The elemental analysis was conducted by energy-dispersive X-ray spectroscopy equipped with the SEM.

Biofunctionalization of AgNPs@MBA@SiO_2

Encoded AgNPs@MBA@SiO_2 were primed with commonly used silane coupling agent GPTMS by mixing 1 mL 4.5×10^{10} NPs/mL AgNPs@MBA@SiO_2 ethanolic solution with 105 μl 0.01% v/v GPTMS ethanolic solution under stirring at 60 °C for 12 h. This amount of GPTMS was calculated to provide approx. GPTMS 20 molecules/nm^2 of AgNPs@MBA@SiO_2 surface. Then GPTMS modified AgNPs@MBA@SiO_2 (AgNPs@MBA@SiO_2@GPTMS) was cleaned by centrifugation at 4000 rpm 8 min with ethanol and phosphate buffered saline (PBS).

This 1 mL AgNPs@MBA@SiO_2@GPTMS was further diluted with PBS into 2.5 mL before biofunctionalization with SARS-CoV/SARS-CoV-2 Spike antibody (Anti-CoV spike Ab, Cat: 40150-D003, MW 150 kDa). Then 2.5 mL of 2 M freshly prepared Ammonium sulphate solution in PBS and 25 μg Anti-CoV spike Ab were added into AgNPs@MBA@SiO_2@GPTMS solution and reaction was kept for 24 h on a rocker table with 110 rpm in a 37 °C room. When immobilization was concluded, final concentration of 0.1% bovine serum albumin (BSA) solution in PBS was added to avoid the adhesion of the NPs to the centrifuge tubes during centrifugation. The Anti-SARS-CoV-2 spike Ab functionalized AgNPs@MBA@SiO_2@GPTMS (AgNPs@MBA@SiO_2@Ab) were cleaned by centrifugation at 4000 rpm 8 min with 0.1% BSA PBS solution three times to eliminate any unbound antibody. AgNPs@MBA@SiO_2@Ab was resuspended into 0.1% BSA PBS solution to further stabilize the NPs in the saline environment with approx. concentration 9×10^9 NPs/mL.

This successful immobilization was confirmed with confocal laser scanning microscope (CLSM) (Leica SP2 (inverted)). 10 μL 9×10^9 NPs/mL AgNPs@MBA@SiO_2@Ab mixed with 15 μL PBS and 25 μL secondary antibody (5 μg/mL, Alexa Fluor 488 AffiniPure Goat Anti-Mouse IgG) for 1 h at room temperature. One negative control was conducted

with AgNPs@MBA@SiO_2 following the same method used for AgNPs@MBA@SiO_2@Ab.

SERS-based biosensing:

Coating plates with capturing Ab: Thawed and mixed by gently vortexing SARS-CoV/SARS-CoV-2 Spike antibody vials (capture Ab, Cat: 40150-D001, MW 150 kDa) before diluting in PBS. 96-well microtiter plates were coated with 50 µl of 6 µg/mL capture Ab per well. The combination between capturing antibody and the plate is created by physical adsorption between the hydrophobic groups of the protein and the plate. To avoid bubbles and ensure homogenous coating on the bottom of every well, lightly taped the plate against hard surface. Plates were sealed with parafilm and aluminium foil to protect from the light and incubated at 4°C overnight.

Blocking plates: Coated plates were washed 3 times with PBS containing 0.1% Tween 20 (PBS-T) solution by adding 300 µL PBS-T solution for each wash. To remove residual buffer, plates were blotted forcefully on a paper towel after each wash. Blocking solution was prepared by mixing BSA into PBS-T solution with concentration of 1%. 200 µl blocking solution was added into each well of the plates and incubated in a 37 °C hot room for one hour. After the blocking incubation, throwed off the blocking solution and tapped the plates dry on a paper towel to remove residuals.

Spike RBD protein assay: Spike RBD protein (SARS-CoV Spike/RBD Protein, MW 26.5 kDa) were diluted with PBS into a series concentration: 0.01, 0.02, 0.1, 0.2, 0.4, 0.6, 0.8, 1, 1.2, 1.6 ng/µL. 50 µl of diluted spike RBD protein (and PBS used as negative control) were transferred into wells in the plate. Plates were placed in a 37 °C hot room for 1h, followed by washing with PBS thrice. Then 50 µl 9×10^9 NPs/mL AgNPs@MBA@SiO_2@Ab solution was added to each working well of the plate. Be sure to avoid touching the walls in order to avoid high background. Plates were incubated in a 37 °C hot room for 1 h, then washed with PBS thrice. Plates were analyzed with Raman by checking SERS signal of labelled AgNPs@MBA@SiO_2@Ab. 5X Leica objective with integration time 1 s and a power at the sample of 50 mW was used for SERS spectra acquisition. Within this set up and for the sake of fast acquisition and result, we collected the SERS signal from randomly selected positions of the plate instead of scanning the whole surface. An optimization of the set up should include reducing the detecting area and measuring with a handheld Raman *in situ*. The intensity at 1075 cm^{-1} were calculated by averaging with 8 spectra from 8 random places in plates.

Limit of detection (LOD)

The limit of detection (LOD) was calculated based on 3 times the signal to noise ratio, by measuring the intensity ratio with the presence and the absence of antigen at 1075 cm^{-1}.

RESULTS AND DISCUSSION

AgNPs@MBA@SiO_2 synthesis and characterization

Figure SI-1 shows a hydrodynamic diameter of 133.8 nm with PDI 0.130 for the AgNPs@MBA@SiO_2 and the zeta potential average of -24.7 mV. This hydrodynamic size agrees to the average size we measured with TEM. And the negative surface charge is typical for the silica outer layer.

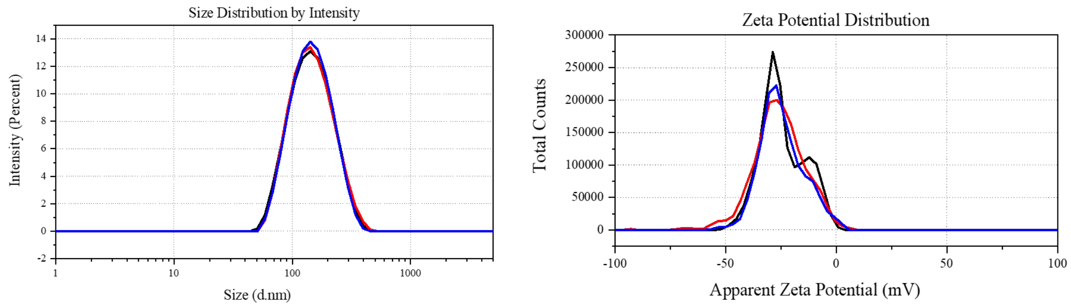

Figure SI-1: Controlled agglomeration and silica coating. **(A)** Size measurements (3 repeated runs) of AgNPs@MBA@SiO_2. **(B)** Zeta potential measurements (3 repeated measurements) of AgNPs@MBA@SiO_2.

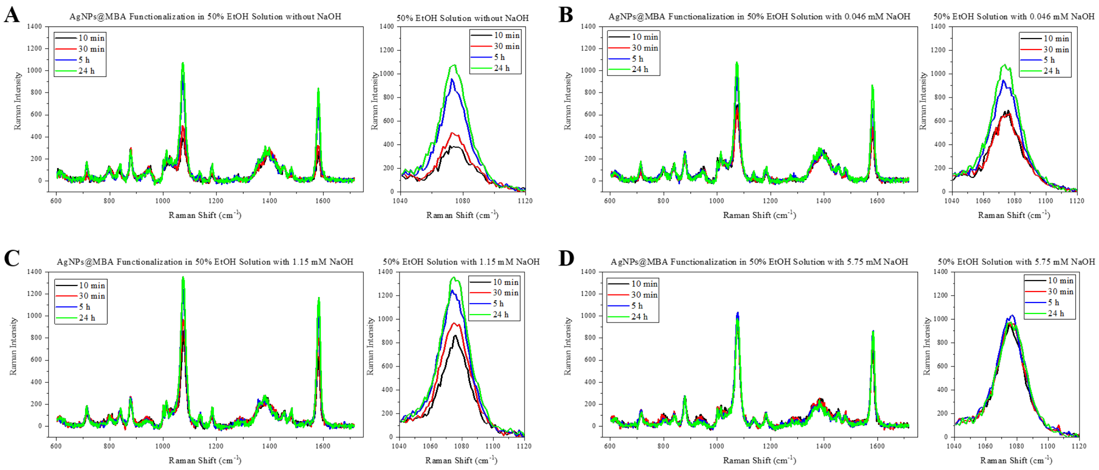

Figure SI-2: SERS spectra and zoomed spectra showing characteristic peak at 1075 cm^{-1} of AgNPs@MBA in 50% EtOH/water solution without NaOH **(A)**, with 0.046 mM NaOH **(B)**, with 1.15 mM NaOH **(C)** and with 5.75 mM NaOH **(D)** at 10 min (in black), 30 min (in red), 5 h (in blue) and 24 h (in green).

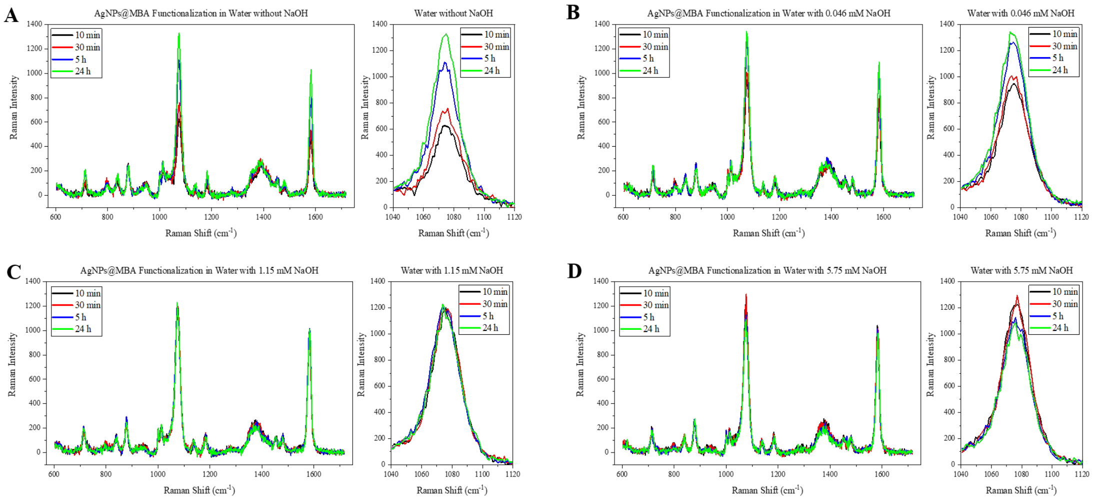

Figure SI-3: SERS spectra and zoomed spectra showing characteristic peak at 1075cm^{-1} of AgNPs@MBA in aqueous solution without NaOH **(A)**, with 0.046 mM NaOH **(B)**, with 1.15 mM NaOH **(C)** and with 5.75 mM NaOH **(D)** at 10 min (in black), 30 min (in red), 5 h (in blue) and 24 h (in green).

**NaOH Amount**	**pH in aqueous solution**	**pH in 50% ethanol/water solution**
without NaOH	6	6.5
0.046 mM NaOH	8.5	6.5
1.15 mM NaOH	11	8
5.75 mM NaOH	11.5	9.5

Table SI-1: pH values for all conditions. The table present the measured pH (test strips) of all codification conditions used for MBA functionalization of AgNPs.

To confirm that we were controlling the degree of agglomeration to achieve optimal optical performance in the AgNPs@MBA@SiO_2, we optically characterized the individual systems (AgNPs and AgNPs@MBA). UV-vis was performed under all codification conditions after 24 h addition of the Raman probe to monitor their LSPR informing on their degree of agglomeration and geometrical changes (figure SI-4). As confirmed, both, AgNPs and AgNPs@MBA, showed the characteristic LSPR of isolated NPs at around 435 nm under all codification conditions. Furthermore, compared with the plamonic absorption of AgNPs, AgNPs@MBA suffered a red shift, around 12 nm, indicating proper adsorption of MBA onto the metallic surface. None of the NPs showed absorption features in the NIR region (approx. 650 nm) attributed to plasmonic coupling of interacting NPs (*i.e.,* agglomeration) as shown for AgNPs@MBA@SiO_2 (figure 1C), confirming the different performance we got based on the kinetic results in Figure 1D and

1E were not related with any geometry changes in AgNPs@MBA.

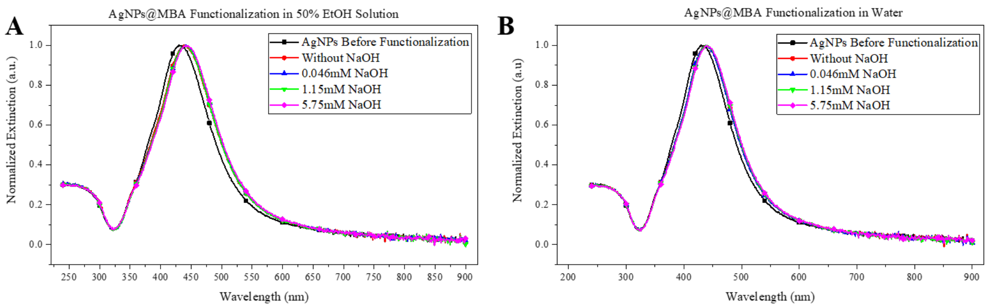

Figure SI-4 UV-Visible extinction spectra of MBA modified AgNPs (AgNPs@MBA) at different pH. Different amounts of NaOH were added to 50% EtOH/water solution **(A)** and to aqueous solution **(B)**. Black line: AgNPs; Red line: AgNPs@MBA without NaOH; Blue line: AgNPs@MBA with 0.046 mM NaOH; Green line: AgNPs@MBA with 1.15 mM NaOH; Magenta line: AgNPs@MBA with 5.75 mM NaOH.

For the controlled agglomeration, we compared 3 nanoparticles with different agglomeration status, *i.e.* non-agglomerated, controlled agglomerated and uncontrolled agglomerated AgNPs@MBA@SiO_2. The SERS intensity of controlled agglomerated nanoparticles at 1075 cm^{-1} was increased close to 40 times compared with non-agglomerated nanoparticles, while uncontrolled nanoparticles have 8 times higher enhancement than non-agglomerated nanoparticles. The degree of agglomeration can be characterized with UV-vis absorption.

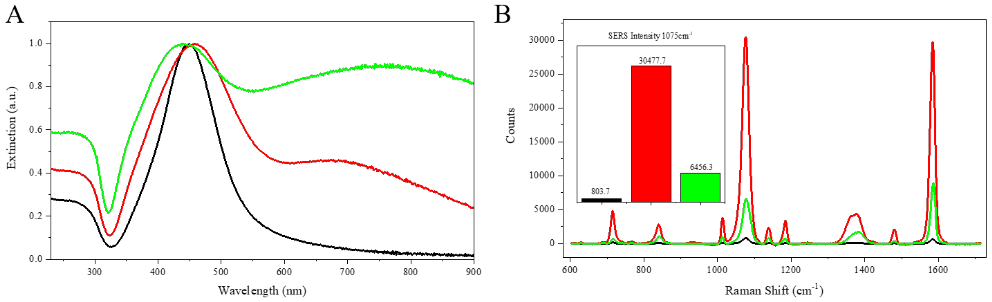

Figure SI-5: UV-vis (A) and Raman (B) characterization for nanoparticles agglomeration. Black lines and column: AgNPs@MBA@SiO_2 non-agglomerated; red lines and column: AgNPs@MBA@SiO_2 agglomerated (controlled agglomeration, the NPs following our protocol); green lines and column: AgNPs@MBA@SiO_2 agglomerated (uncontrolled agglomeration; with high centrifuge speed and time (4800 rpm (2000 g) 20 min, twice)).

AuNPs are synthesized following the method reported as sodium citrate method [1]. Then we performed the procedure described before for the Ag-based nanoagglomerates *i.e.*, codification with MBA and stabilization with CTPEG12, controlled agglomeration and the SiO_2 encapsulation. The complete characterization is presented in Figure SI-6. Figure

SI-6A shows the TEM image of AuNPs@MBA@SiO_2. The AuNPs were spherical and more than 60% of the NPs were agglomerated (measured from 100 NPs from TEM images). Figure SI-6B exhibit the size histogram of AuNPs@MBA@SiO_2 showing an average diameter of around 107 nm. This value was obtained by measuring 4 different angles of 100 NPs containing all populations (isolated NPs, dimers, trimers, tetramers, pentamers, and hexamers). Figure SI-6C shows that the AgNPs@MBA@SiO_2 have a hydrodynamic diameter of 113.6 nm with PDI 0.199, and a zeta potential average of -27.9 mV (figure SI-6D). The normalized extinction spectra of AuNPs and AuNPs@MBA@SiO_2 is presented in figure SI-6E. Spherical, isolated AuNPs have their characteristic LSPR peat at around 540 nm whereas the AuNPs@MBA@SiO_2 exhibited the characteristic absorption feature attributed to agglomerated NPs which is shifted to the NIR region, in this case to approx. 750 nm. The SERS spectra and the peak intensity at 1075 cm^{-1} of AuNPs@MBA@SiO_2 and non-agglomerated AuNPs@MBA@SiO_2 is presented in figure SI-6F. It demonstrates controlled agglomeration and adsorption of the Raman probe to increase the SERS efficiency (2 times).

Au-based nanoagglomerates present similar physicochemical characteristics (size, surface charge, polydispersity, agglomerates form) than Ag-based. The LSPR were different because it is related to composition of the plasmonic nanostructure (Ag *vs* Au). This demonstrates the reproducibility and robustness of our synthesis procedure. We did observe differences in the SERS performance. AuNPs@MBA@SiO_2 nanoagglomerates enhanced 2 times (figure SI-6F) whereas AgNPs@MBA@SiO_2 nanoagglomerates achieved almost 40 times higher signals (figure 1F) than their non-agglomerated counterparts.

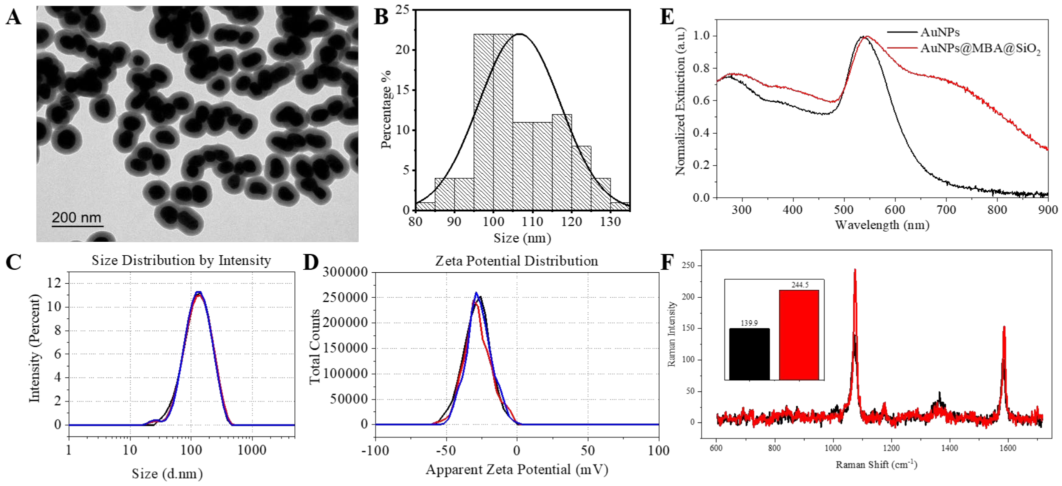

Figure SI-6: Synthesis and characterization of AuNPs@MBA@SiO₂. (A) TEM image of AuNPs@MBA@SiO2. **(B)** Size distribution of AuNPs@MBA@SiO2 based on TEM images of 100 NPs analyzed with ¨Image J¨. **(C)** Size measurements (3 repeated runs) of AuNPs@MBA@SiO2, with mean value 113.6 nm and PDI 0.199; **(D)** Zeta potential measurements (3 repeated measurements) of AuNPs@MBA@SiO2, with mean value -27.9 mV. **(E)** UV-Visible extinction spectra of AuNPs (in black) and AuNPs@MBA@SiO2 (in red). **(F)** SERS spectra and SERS intensity at 1075 cm^{-1} (inset image) of non-agglomerated AuNPs@MBA@SiO2 (in black) and AuNPs@MBA@SiO2 (in red).

Stability of the AgNPs@MBA@SiO₂ SERS signal after labelling different types of substrates

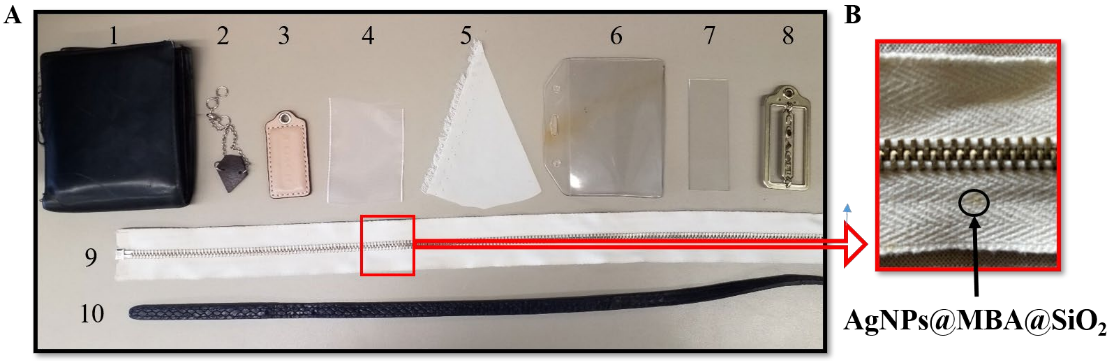

Figure SI-7: Measuring the stability of the SERS signal onto different materials. (A) Picture showing 10 substrates labelled with AgNPs@MBA@SiO_2. **(B)** zoomed photo showing AgNPs@MBA@SiO_2 deposited on cotton. The materials are: 1, semi-aniline leather; 2, aniline leather; 3, pigmented leather; 4, polyester; 5, silk; 6, plastic (PVC); 7, glass; 8, brass; 9, cotton; and 10, dyed pigmented leather.

COVID-19 SERS-based diagnosis

To confirm NPs' surface bioconjugation, we incubate the AgNPs@MBA@SiO_2@Ab and the AgNPs@MBA@SiO_2 with a fluorescently labelled secondary antibody. Only AgNPs@MBA@SiO_2@Ab provide a bright fluorescent signal originated from the antibody recognition and confirming successful bioconjugation (figure SI-8).

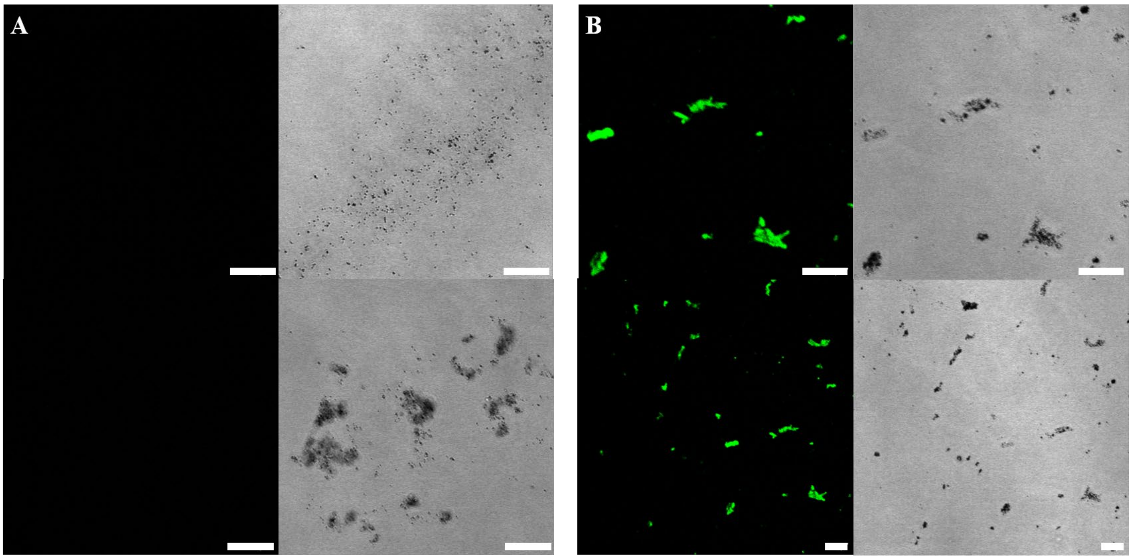

Figure SI-8: Synthesis and characterization of AgNPs@MBA@SiO_2@Ab. Confocal laser scanning microscopy images of AgNPs@MBA@SiO_2 **(A)** and AgNPs@MBA@SiO_2@Ab **(B)** incubated with a fluorescently labeled secondary antibody (Alexa Fluor 488 AffiniPure Goat Anti-Mouse IgG) recognizing the antibody on the surface of the NPs. The scale bar in all confocal images correspond to 10 µm.

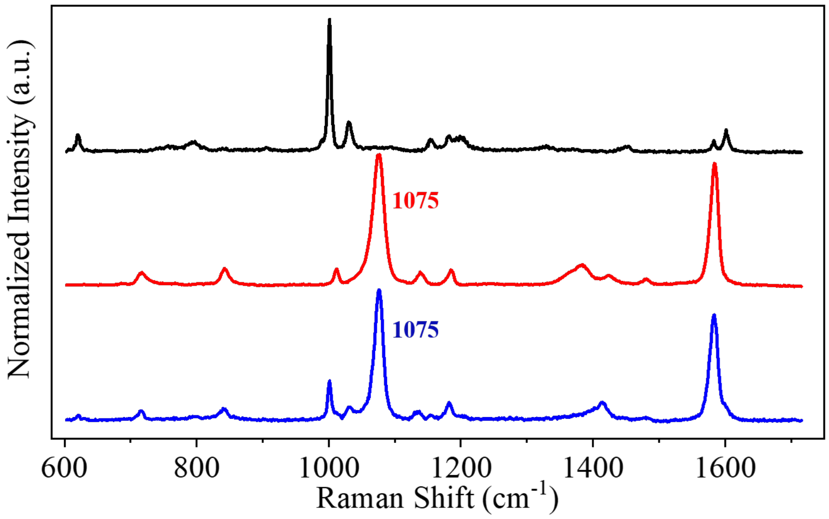

Figure SI-9: SERS response of the different components. The black spectrum corresponds to the plate (composed of polystyrene). The red spectrum was recorded from the AgNPs@MBA@SiO_2@Ab in buffer and showing the characteristic MBA peak at 1075 cm^{-1}. The blue spectrum corresponds to the recognition of 1 ng/μL SARS-CoV-2 spike RBD protein immobilized onto a plate by our AgNPs@MBA@SiO_2@Ab. The appearance of the at 1075 cm^{-1} confirms virus detection.

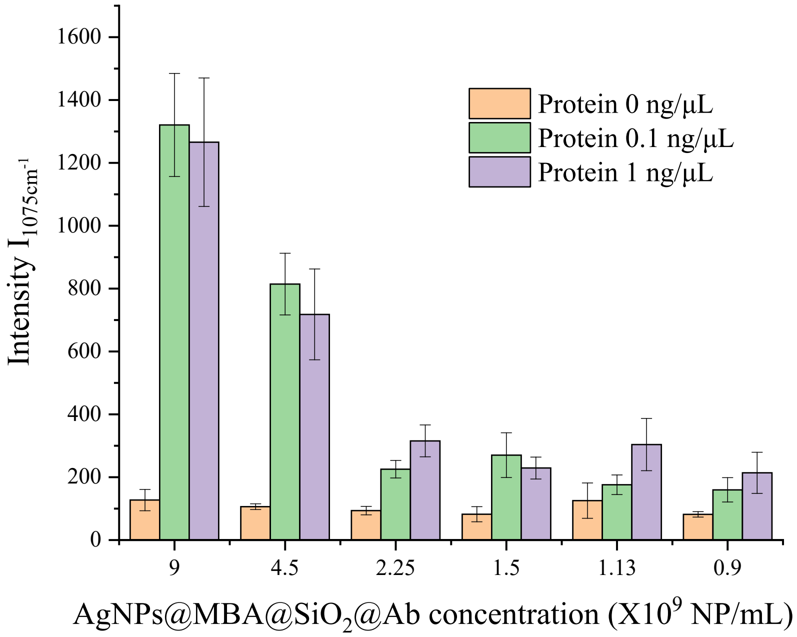

Figure SI-10: AgNPs@MBA@SiO_2@Ab concentration analysis for detection. The concentration effect of AgNPs@MBA@SiO_2@Ab on SARS-CoV spike RBD protein detection were exploited. 0, 0.1 and 1 ng/μL SARS-CoV spike RBD protein were tested. The averages and their standard deviation were analyzed with 8 spectra which were

collected from 8 random positions on the well (Spectra were analyzed directly without baseline subtraction).

Targeted markers	Sensing principle	Materials	LOD	Reference
S1 spike protein	Bioelectric Recognition Assay	Membrane-engineered mammalian cells	1 fg/mL	[2]
S1 spike protein	Immunoassay-electrical transduction	Bilayer epitaxial graphene	1 ag/mL	[3]
S1 spike protein and spike RBD	Lateral flow immunoassay	Cellulose nanobeads	<5 ng/reaction (100µL) spike protein <1 ng/reaction (100µL) spike RBD	[4]
Spike protein	Bioelectronic sensor	Vertically-oriented silicon nanowire arrays	100 ng/ml or 575 pmol/L	[5]
Spike RBD	LSPR sensor	Silver nanotriangle array	0.83 pmol/L	[6]
Spike RBD	Fluorescence resonance energy transfer system	hACE2 mimic peptide-based molecular beacon	52.5 pmol	[7]
Spike RBD	Near-infrared fluorescence	Single-walled carbon nanotube	12.6 nmol/L	[8]
Spike RBD	SERS-immunoassay	Silver silica nanocomposites	<0.01 ng/µL (0.5 ng/reaction)	This work

Table SI-2: Comparison table of different SARS-CoV-2 spike protein detection assays. The columns from the left to the right show the biomarker detected, the readout, the material composition, limit of detection (LOD) and the corresponding references.

REFERENCES

1. Turkevich J, Stevenson PC, Hillier J. A study of the nucleation and growth processes in the synthesis of colloidal gold. *Discuss Faraday Soc*. **11**, 55-75 (1951).

2. Mavrikou S, Moschopoulou G, Tsekouras V, Kintzios S. Development of a Portable, Ultra-Rapid and Ultra-Sensitive Cell-Based Biosensor for the Direct Detection of the SARS-CoV-2 S1 Spike Protein Antigen. *Sensors*. **20**, 3121 (2020).

3. Kim S, Ryu H, Tai S, et al. Real-time ultra-sensitive detection of SARS-CoV-2 by quasi-freestanding epitaxial graphene-based biosensor. *Biosens Bioelectron*. **197**, 113803 (2022).

4. Lee JH, Choi M, Jung Y, et al. A novel rapid detection for SARS-CoV-2 spike 1 antigens using human angiotensin converting enzyme 2 (ACE2). *Biosens Bioelectron*. **171**, 112715 (2021).

5. Gao B, Rojas Chavez AA, Malkawi WI, et al. Sensitive detection of SARS-CoV-2 spike protein using vertically-oriented silicon nanowire array-based biosensor. *Sens Bio-Sensing Res*. **36**, 100487 (2022).

6. Yang Y, Murray J, Haverstick J, Tripp RA, Zhao Y. Silver nanotriangle array based LSPR sensor for rapid coronavirus detection. *Sensors Actuators B Chem*. **359**, 131604 (2022).

7. Kang B, Lee Y, Lim J, et al. FRET-Based haCE2 Receptor Mimic Peptide Conjugated Nanoprobe for Simple Detection of SARS-CoV-2. *Chem Eng J*. **442**, 136143 (2022).

8. Pinals RL, Ledesma F, Yang D, et al. Rapid SARS-CoV-2 Spike Protein Detection by Carbon Nanotube-Based Near-Infrared Nanosensors. *Nano Lett*. **21**(5), 2272-2280 (2021).
